# Impact of the Choosing Wisely Canada recommendations on potentially inappropriate antibiotic prescribing in emergency medicine across Alberta, Canada: An interrupted time-series analysis.

**DOI:** 10.23889/ijpds.v7i3.1850

**Published:** 2022-08-25

**Authors:** Jason Black, David Campbell, Kerry McBrien, Paul Ronksley, Eddy Lang, Tyler Williamson

**Affiliations:** 1 University of Calgary

## Objectives

In Alberta, Canada, we quantified the rate of potentially inappropriate oral antibiotic prescribing in emergency departments for viral infections or conditions not likely requiring antibiotics from 2010-2020 and assessed the impact of two Choosing Wisely Canada (CWC) campaigns (2015/2016 and 2018) discouraging inappropriate antibiotic prescribing in emergency medicine.

## Approach

We linked emergency department adult and pediatric records from the National Ambulatory Care Reporting System and medication dispensations from community-based pharmacies in the Pharmaceutical Information Network. From January 2010 to February 2020, we identified emergency department visits for 5 conditions that were potentially inappropriately treated using antibiotics per CWC recommendations (bronchitis, asthma, bronchiolitis, pharyngitis, and acute otitis media). We used an interrupted time series design to detect changes in antibiotic prescribing by fitting Autoregressive Integrated Moving Average (ARIMA) models to account for secular trends and seasonality, allowing for change in slopes to measure the effect of each CWC intervention.

## Results

Antibiotics were commonly prescribed in emergency departments for bronchitis (proportion of visits with antibiotics: 57%) and asthma (22%) in adults; bronchiolitis in children (43%); pharyngitis (39%) and acute otitis media (54%) in adults and children. Based on visual inspection, the proportion of emergency department visits for each condition where antibiotics were dispensed remained relatively consistent. The ARIMA models demonstrated mixed impacts on potentially inappropriate antibiotic prescribing associated with two interruptions: the 2015/2016 CWC recommendations and subsequent 2018 CWC Using Antibiotics Wisely campaign. Following each interruption, antibiotic prescribing was slightly reduced for bronchitis (-1.0%/year,p=0.03; -4.4%/year,p=0.004, respectively) and bronchiolitis (not significant) (-0.7%/year,p=0.57; -2.5%/year,p=0.34), but unchanged for asthma (-0.6%/year,p=0.30; 0.7%/year,p=0.74) and pharyngitis (0.0%/year,p=0.95; -0.2%/year,p=0.93), and slightly increased for acute otitis media (not significant) (1.4%/year,p=0.07; 5.9%/year,p=0.052).

## Conclusion

Rates of potentially inappropriate antibiotic prescribing remained constant over the past 10 years in Alberta. Campaigns to rethink antibiotic use in emergency medicine may have resulted in small decreases in antibiotic use; however, further initiatives building upon existing campaigns are required to substantially reduce rates of inappropriate antibiotic prescribing.

